# Changes in coverage among non-elderly adults with chronic diseases following Affordable Care Act implementation

**DOI:** 10.1371/journal.pone.0278414

**Published:** 2022-11-30

**Authors:** John D. Goodson, Sara Shahbazi, Zirui Song

**Affiliations:** 1 Division of General Internal Medicine, Department of Medicine, Massachusetts General Hospital, Boston, Massachusetts, United States of America; 2 Department of Health Care Policy, Harvard Medical School, Boston, Massachusetts, United States of America; Kasturba Medical College Mangalore, Manipal Academy of Higher Education, INDIA

## Abstract

**Importance:**

Changes in insurance coverage after the Affordable Care Act (ACA) among non-elderly adults with self-reported chronic conditions across income categories have not been described.

**Objective:**

To examine changes in insurance coverage after the ACA among non-elderly adults with chronic conditions across income categories, by geographic region.

**Design:**

We compared self-reported access to health insurance pre-ACA (2010-2013) and post-ACA (2014-2017) for individuals 18-64 years of age with ≥ 2 chronic conditions, including hypertension, heart disease/stroke, emphysema, diabetes, asthma, cancer, and arthritis, across regions using a logistic regression approach, adjusted for covariates. We also assessed U.S. Census regional differences in insurance coverage post-ACA using modified Poisson regression models with robust variance and calculated the risk ratio (RR) of being uninsured by region, with the Northeast as the reference category. Within each region, we then examined changes in insurance coverage by income level among non-elderly individuals with any chronic condition.

**Setting:**

2010–2017 household component of the nationally representative Medical Expenditure Panel Survey (MEPS).

**Participants:**

All members of surveyed households during five interviews over a two-year period.

**Intervention:**

Start of insurance coverage expansion under the ACA.

**Main outcomes:**

Health insurance status.

**Results:**

On average nationwide, non-elderly adults with self-reported chronic conditions experienced increased insurance coverage associated with the ACA (diabetes: +6.41%, high-blood pressure: +6.09%, heart disease: +6.50%, asthma: +6.37%, arthritis: +6.77%, and ≥ 2 chronic conditions: +6.39%). Individuals in the West region reported the largest increases (diabetes +9.71%, high blood pressure +8.10%, and heart disease/stroke +8.83 %, asthma +9.10%, arthritis +8.39%, and ≥ 2 chronic conditions +8.58). In contrast, individuals in the South region reported smaller increases in insurance coverage post-ACA among those with diabetes, heart disease/stroke, and asthma compared to the Midwest and West. The Northeast region, which had the highest levels of insurance coverage pre-ACA, exhibited the smallest increase in reported coverage post-ACA. Reported insurance coverage improved across all regions for adults with any chronic condition across income levels, most notably for very low- and low-income individuals. A further cross-sectional comparison after the ACA demonstrated important residual differences in insurance coverage, despite the gains in all regions. When compared to the Northeast, adults with any self-reported chronic conditions living in the South were more likely to report no insurance coverage (diabetes: RR 1.99, p-value <0.001, high blood pressure: RR 2.02, p-value <0.001, heart diseases/stroke: RR 2.55, p-value <0.001, asthma RR 2.21, p-value <0.001, arthritis: RR 2.25, p-value <0.001), and ≥ 2 chronic condition (RR 2.29, p-value <0.001).

**Conclusion and relevance:**

The ACA was associated with meaningful increases in insurance coverage for adults with any self-reported chronic condition in all US regions, most notably in the West region and among those with lower incomes, suggesting a nation-wide trend to improved access to health insurance following implementation. However, intra-regional comparisons after ACA implementation showed important differences. Individuals with ≥2 chronic conditions in the South were 2.29 times less likely to have insurance coverage in comparison to their peers in the Northeast. Though the post-ACA improvements in reported access to health insurance coverage affected all US regions, the reported experience of those with multiple chronic conditions in the South point to continued barriers for those most likely to benefit from access to health insurance coverage. Medicaid expansion in the South would likely result in increased insurance coverage for individuals with chronic conditions and improve health care outcomes.

## Introduction

The Affordable Care Act (ACA) expanded health insurance coverage to roughly 20 million individuals in the U.S. through two key mechanisms: the ACA Marketplace with subsidies for individuals to purchase private plans and Medicaid expansions [1,2]. Recently, the Assistant Secretary for Planning and Evaluation (ASPE) assessed the impact of the ACA on various segments of the population based on location, gender, disability, membership in an American Indian tribe, immigrant status, and sexual orientation [3]. However, to date there has not been a similar assessment of the ACA’s impact on individuals with self-reported chronic conditions. In this paper, we assessed changes in insurance coverage reported by U.S. adults with self-reported chronic conditions from before to after the ACA.

Prior to the ACA’s coverage expansion, the Department of Health and Human Services (HHS) found that 50 to 129 million non-elderly Americans had some type of pre-existing health condition. Notably, one in five non-elderly Americans reporting a pre-existing condition—25 million individuals—were uninsured. Of those with pre-existing conditions who sought private insurance, 47% could not get comprehensive coverage [4]. These individuals were generally denied coverage, charged higher premiums, or had their preexisting condition(s) excluded from coverage. The ACA prevented those with preexisting conditions from being denied coverage or charged higher premiums.

The prevalence of chronic diseases varies geographically in the U.S. due in part to differences in demographics and health behaviors [5]. Evidence suggests that the prevalence of adults with multiple chronic conditions has been highest in the East, South, Central, and Middle Atlantic regions of the country [6]. State approaches to adopting the ACA’s provisions have varied significantly based on state statutes, the state’s political environment, and economic factors. As a consequence, the bulk of Medicaid expansion was driven by a subset of states [7]. After the first two years of ACA implementation (2014-16), Medicaid was expanded in nearly all states except those in the South US Census region, despite the higher prevalence of chronic conditions in that region. In addition to protections for preexisting conditions and Medicaid expansion targeted to low-income Americans, the ACA Marketplace expanded access to commercial insurance coverage by providing subsidies to individuals and families with incomes above the Medicaid eligibility threshold.

Within a nationally representative sample of non-elderly adults with self-reported chronic conditions, we examined changes across four US regions in insurance access after ACA implementation for non-elderly individuals reporting diabetes, high blood pressure, heart disease/stroke, asthma, arthritis, and ≥2 chronic conditions. We then compared the post-ACA intra-regional reported experiences for non-elderly individuals with any chronic condition by income for non-elderly individuals across four income brackets.

Our analyses address the following questions: (1) How did self-reported health insurance coverage among non-elderly individuals with chronic conditions change across the four US regions after ACA implementation? (2) How did insurance access compare among the US regions after ACA implementation for non-elderly individuals with any chronic condition across income brackets?

## Methods

### Data

We used the household component of the Medical Expenditure Panel Survey (MEPS) for 2010–2017. The MEPS is a nationally representative sample of the nonmilitary, noninstitutionalized population. The survey collects information from a household reference person concerning insurance status, use of health care services, and health care spending for each individual member of a surveyed household through five interviews over a 2-year period [8]. The MEPS 2-year overlapping panel design facilitates the combination of data from 2 panels to obtain data from each year (e.g., data for 2015 combine the overlapping panels of 2014–2015 and 2015–2016). Each year of MEPS data is designed to be nationally representative, and pooling the data produces average annual estimates [9]. The MEPS average response rate for the 2010-2017 Full-Year Files was 55.2% [10]. Eight years of cross-sectional data were pooled to maximize sample size. Since most provisions of the ACA took effect beginning in 2014, we defined MEPS responses 2010-2013 as the pre-ACA cohort and 2014-2017 as the post-ACA cohort. Since we analyzed a subset of MEPS data, we were not able to reliably track trends over time in the cohorts. Likewise, MEPS data are not sufficient to determine the impact on the ACA on a state-by-state basis for the subsets of interest, those with chronic conditions and those in different income brackets. Therefore, our comparisons were among US Census regions. This study was approved by the IRB of the Massachusetts General Hospital, Boston.

### Measures

MEPS offers a unique view of how individuals see their health and access to insurance. There is no validation of the health conditions reported nor are there in home clinical assessments. MEPS “priority” chronic conditions are determined through a series of questions about whether a physician or other health care professional ever informed the person about the presence of a specified diagnosis. We extracted records for individuals aged 18-64 years with at least one of the MEPS chronic conditions (excluding attention deficit disorders): high blood pressure, heart disease/stroke, emphysema, chronic bronchitis, high cholesterol, diabetes, asthma, cancer, joint pain, and arthritis. Multiple chronic conditions were defined as a survey participant reporting ever having been diagnosed with 2 or more of the MEPS chronic conditions (excluding attention deficit disorders). The key outcome variable was health insurance access as reported by the respondent for each household individual. The key independent variable was an indicator for the post-ACA period.

To guide the selection of covariates that could affect insurance access, we relied on the Andersen behavioral model of health services utilization conceptual framework. This model divides the individual factors that influence health services utilization into the following three categories: predisposing, enabling, and need factors. The predisposing factors are socio-demographic characteristics. The enabling factors include personal, family, and community resources that can either facilitate or impede the use of services. Need factors refer to the health conditions—either perceived or evaluated—requiring medical care [11]. In our study, predisposing covariates consisted of sex, age, race, family income as percent of the Federal Poverty Level (FPL), education (no high school degree, high school, bachelor’s degree, advanced degree). Enabling covariates consisted of employment situation (employed, unemployed) and smoking status. Under the ACA, some group health plans, and self-insured employers, can charge tobacco users up to 50% more for their health insurance premiums than non-tobacco users through a tobacco surcharge, thus, smoking status can impact individuals’ decision on obtaining an insurance [12]. Need covariates consisted of perceived physical and mental health status, and having a usual source of care.

Family income categories were extracted from the MEPS poverty status variable, which classifies income based on the percentage of the federal poverty level (FPL) for total family income, adjusted for family size and composition. We classified “family income level” into four levels: very low income (< 125% FPL), low income (125% to < 200% FPL), middle income (200% to < 400% FPL), and high income (≥ 400% FPL). The measures of perceived physical and mental health status were constructed by MEPS using the question, “how one thinks of one’s health relative to the health of people in one’s age group,” and responses were scored on a 5-item scale: 1) excellent, 2) very good, 3) good, 4) fair, and 5) poor. Region of residence was a categorical variable based on four US Census regions: Northeast, Midwest, South, and West.

### Statistical analyses

To assess changes in reported coverage associated with the ACA, we compared the pre-ACA period (2010-2013) with the post-ACA period (2014-2017). First, we tabulated the demographic and clinical characteristics of the study population, stratified by U.S. Census region. Second, within each region and among non-elderly adults with a subset of chronic conditions (diabetes, high blood pressure, heart disease/stroke, asthma, and arthritis), we used logistic regression models to test the associations between the implementation of the ACA coverage expansions and insurance coverage, adjusted for the study covariates. Third, within each region, we examined changes in insurance coverage by income level among individuals with at least one chronic condition. To obtain percentage point changes in insurance coverage associated with the ACA, we calculated predictive marginal effects. Specifically, we used STATA’s “margins” command to estimate absolute percentage point differences, as opposed to odds ratios, to facilitate interpretation of results. Fourth, for each chronic condition above, we applied modified Poisson regression models with robust variance to examine regional differences among the four regions in insurance status post-ACA for estimating relative risks [13]. We used the Northeast as the reference category, as this region had the highest baseline insurance coverage rate. Associations were presented as risk ratios (RR), with statistical significance was defined as a p-value < 0.05.

## Results

### Characteristics of the population

Table 1 presents the MEPS population demographic and clinical characteristics by region. The South region had a higher percentage of African-American population (31.39% vs. Northeast 22.25%, Midwest 17.70%, and West 6.21%), very low family income (28.08% vs. Northeast 23.89%, Midwest 21.70%, and West 22.46%), diabetes (8.79% vs. Northeast 6.96%, Midwest 7.36%, and West 6.63%), and high blood pressure, (28.58% vs. Northeast 24.33%, Midwest 25.90%, and West 20.33%). The Midwest showed a higher percentage of arthritis (20.05% vs. Northeast 18.01%, South 17.80%, and West 12.88%) and current smoking (22.97% vs. Northeast 17.44%, South 19.72%, and West 11.83%). The West region has the highest rate of uncompleted high school education compared to other regions (24.86% vs. Northeast 18.96%, Midwest 16.59%, and South 21.76%) (Table 1).

**Table 1:** Characteristics of the study population by Region, MEPS, 2010-2017.

	Northeast	Midwest	South	West
**Age (mean)**	40.68	40.23	40.05	39.38
**Sex (%)**				
Male	46.49	47.79	46.44	48.10
Female	53.51	52.21	53.56	51.90
**Race (%)**				
White	65.89	74.06	61.80	74.53
Black	22.25	17.70	31.39	6.21
Other	11.86	8.24	6.82	19.26
**Education (%)**				
No degree	18.96	16.59	21.76	24.86
HS, GED	46.32	48.79	49.74	42.67
Bachelor’s	16.91	16.88	13.62	16.31
Postgraduate	9.25	7.94	6.78	7.71
Other	8.56	9.80	8.11	8.44
**Family income (% FPL) (%)**				
Very low-income	23.89	21.70	28.08	22.46
Low income	14.81	14.46	17.27	16.77
Middle income	27.38	31.95	29.79	30.12
High Income	33.91	31.89	24.86	30.64
**Employment (%)**				
Employed	71.60	76.87	71.89	74.04
Not employed	28.40	23.13	28.11	25.96
Perceived physical health status				
Excellent	26.68	23.40	26.32	26.83
Very good	31.55	34.36	30.42	32.81
Good	28.91	29.38	29.25	28.52
Fair/poor	12.86	12.86	14.01	11.84
Perceived mental health status				
Excellent	35.94	33.54	39.05	38.27
Very good	28.95	31.26	26.89	30.35
Good	26.65	26.92	26.29	24.65
Fair/poor	8.46	8.28	7.77	6.73
**Smoking (%)**	17.44	22.97	19.72	11.83
**Diabetes (%)**	6.96	7.36	8.79	6.63
**High blood pressure (%)**	24.33	25.90	28.58	20.33
**Heart disease, stroke (%)**	9.97	11.45	10.21	7.42
**Arthritis (%)**	18.01	20.05	17.80	12.88
**≥ 2 chronic conditions (%)**	31.30	34.89	31.54	26.67

Note. Percentages may not add up to 100 due to missing values. Pre-ACA is an indicator for the years 2010-2013. Post-ACA is an indicator for the years 2014-2017. MEPS= Medical Expenditure Panel Survey; ACA: Affordable Care Act.

### Pre-Post ACA changes in insurance coverage

Table 2 illustrates the pre/post-ACA changes in reported insurance coverage by region for individuals with diabetes, high blood pressure, heart disease/stroke, asthma, arthritis and ≥2 chronic conditions. We found that on average, non-elderly individuals with any of the select chronic conditions reported improved access to health insurance coverage after ACA implementation, (diabetes: +6.41%, high-blood pressure: +6.09%, heart disease: + 6.50%, asthma: +6.37%, arthritis: +6.77%, ≥2 chronic conditions: +6.39%). Regional differences were found in the effect of ACA on insurance status for patients with all the chronic conditions. In the West, individuals with a one or more of the select chronic conditions had the highest increase in reported insurance coverage post-ACA (diabetes +9.71%, high blood pressure +8.10%, and heart disease/stroke +8.83%, asthma +9.10%, arthritis +8.39%, and ≥ 2 conditions +8.58%). The South region showed a much lower reported increase in health insurance access post-ACA for individuals with diabetes, heart disease/stroke, and asthma compared to Midwest and West (diabetes: +4.84% vs Midwest +9.03%, West +9.71%; heart disease/stroke: +5.11% vs Midwest +7.73% and West +8.83%; and asthma: +5.17% vs. Midwest +6.53% and West +9.10%).

**Table 2:** The Impact of ACA on Insurance Status of Individuals Ages 18-64 with each select Chronic Condition by US Census Region, MEPS Survey Data 2010-2017.

Share of the Population with Insurance Coverage					
	Unadjusted			Adjusted Change	
	Pre-ACA	Post-ACA	Percentage Point Change	Percentage Point Change	p-value
**Diabetes**					
Northeast	89.73	92.47	+2.74	+2.15	0.141
Midwest	83.38	92.00	+8.62	+9.03	<0.001
South	77.30	83.15	+5.85	+4.84	<0.001
West	77.95	88.50	+10.55	+9.71	<0.001
**High blood pressure**					
Northeast	88.56	92.13	+3.57	+3.64	<0.001
Midwest	83.86	90.96	+7.10	+6.01	<0.001
South	76.20	82.34	+6.14	+5.98	<0.001
West	80.12	88.69	+8.57	+8.10	<0.001
**Heart disease/ stroke**					
Northeast	89.97	93.87	+3.9	+3.96	<0.001
Midwest	83.70	91.35	+7.65	+7.73	<0.001
South	78.59	84.39	+5.8	+5.11	<0.001
West	82.75	92.31	+9.56	+8.83	<0.001
**Asthma**					
Northeast	89.28	93.46	+4.18	+4.51	<0.001
Midwest	84.39	91.98	+7.59	+6.53	<0.001
South	77.13	84.97	+7.84	+5.17	<0.001
West	82.7	92.8	+10.1	+9.10	<0.001
**Arthritis**					
Northeast	90.83	94.69	+3.86	+3.34	<0.001
Midwest	85.93	93.22	+7.29	+7.29	<0.001
South	79.33	86.21	+6.88	+6.61	<0.001
West	84.43	93.42	+8.99	+8.39	<0.001
**≥ 2 chronic conditions**					
Northeast	88.70	92.35	+3.64	+3.75	<0.001
Midwest	82.94	90.47	+7.52	+7.02	<0.001
South	76.42	82.99	+6.57	+5.52	<0.001
West	80.79	89.96	+9.17	+8.58	<0.001

Note: We used separate logistic regression models for each chronic condition and comorbidity adjusted for sex, age, race, family income, education, smoking status, employment status, perceived physical and mental health status, and having a usual source of care and present the percentage-point change in insurance coverage.

### Intra-regional comparisons of insurance access post-ACA

We the compared the intra-regional differences of insurance access after ACA implementation. Using the Northeast U.S. region as the reference, we found that the risk of being uninsured was highest in the South region for all those with each of the chronic conditions, Table 3. Compared to the NE, non-elderly adults with each of the chronic conditions living in the South were more likely to be uninsured post-ACA (diabetes: RR 1.99, p-value <0.001; high blood pressure: RR 2.02, p-value <0.001; heart diseases/stroke: RR 2.55; p-value <0.001, asthma: RR 2.21, p-value <0.001; arthritis: RR 2.25, p-value <0.001; ≥ 2 chronic conditions: RR 2.29, p-value <0.001).

**Table 3:** Likelihood of being uninsured post-ACA by US Census Region for individuals ages 18-64 with each select chronic condition, MEPS survey data 2010-2017.

Reported Uninsurance				
	Unadjusted		Adjusted	
	Risk Ratio	P-value	Risk Ratio	p-value
**Diabetes**				
Northeast	Reference			
Midwest	1.06	0.689	0.94	0.740
South	2.24	<0.001	1.99	<0.001
West	1.53	0.002	1.13	0.415
**High blood pressure**				
Northeast	Reference			
Midwest	1.15	0.079	1.18	0.069
South	2.24	<0.001	2.02	<0.001
West	1.44	<0.001	1.31	0.002
**Heart disease/ stroke**				
Northeast	Reference			
Midwest	1.41	0.008	1.40	0.039
South	2.55	<0.001	2.55	<0.001
West	1.25	0.091	1.29	0.122
**Asthma**				
Northeast	Reference			
Midwest	1.23	0.101	1.16	0.318
South	2.30	<0.001	2.21	<0.001
West	1.10	0.448	1.02	0.888
**Arthritis**				
Northeast	Reference			
Midwest	1.28	0.023	1.12	0.370
South	2.60	<0.001	2.25	<0.001
West	1.24	0.051	1.05	0.701
**≥ 2 chronic conditions**				
Northeast	Reference			
Midwest	1.25	0.001	1.20	0.055
South	2.22	<0.001	2.29	<0.001
West	1.31	0.001	1.26	0.013

Note: This table assesses the likelihood of uninsurance across regions post-ACA, using the Northeast region, as the reference group. We used separate logistic regression models for each chronic condition and comorbidity adjusted for sex, age, race, family income, education, smoking status, employment status, perceived physical and mental health status, and having a usual source of care, which produced estimates of the risk ratio. The risk ratio is the ratio of risk of uninsurance in the Midwest, West, and South region versus the Northeast region, post-ACA.

### Post-ACA insurance coverage regional comparison by income

**Fig 1:**
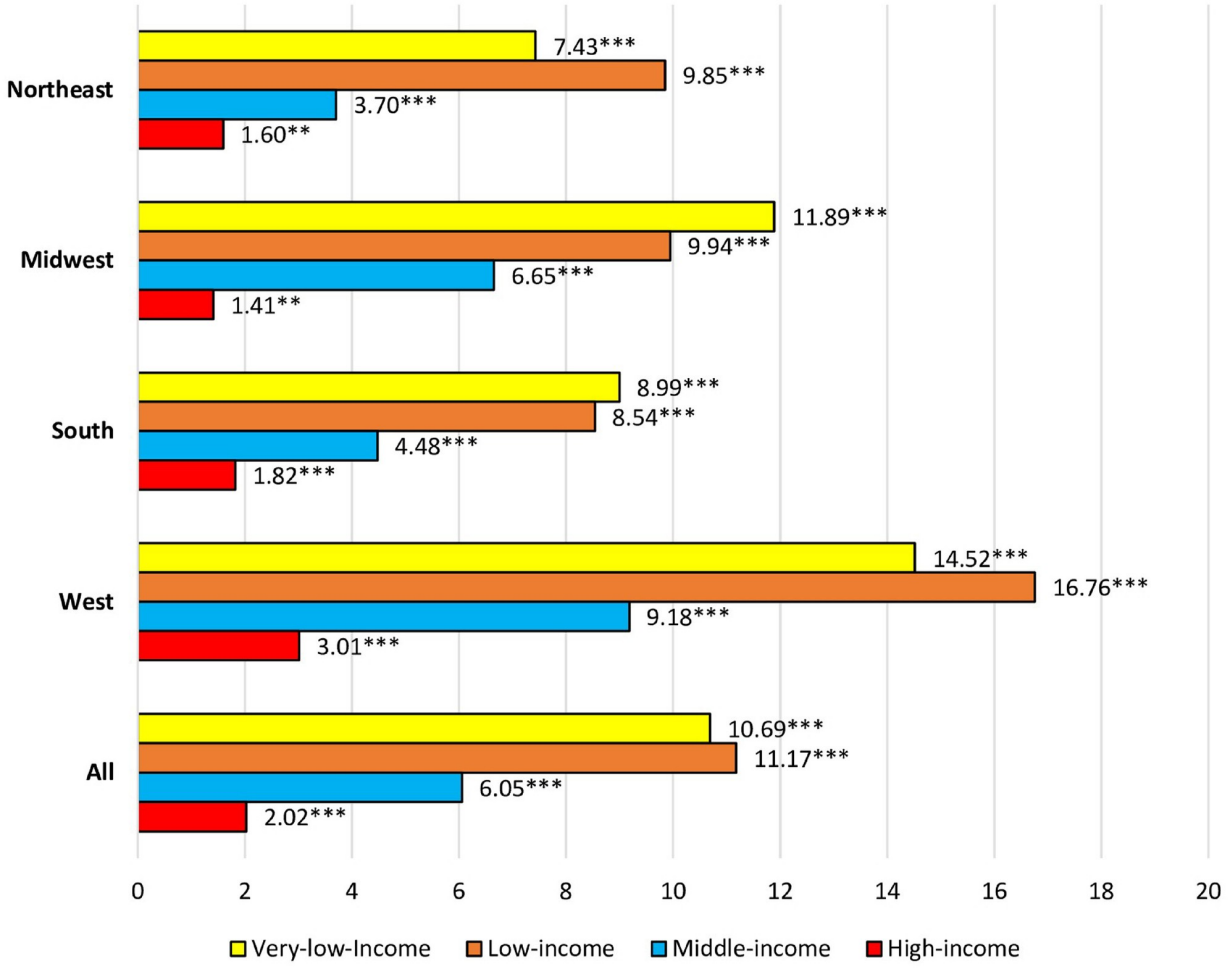
Adjusted percentage point changes in insurance status of individuals ages 18-64 with any Chronic Condition pre-vs. post-ACA by US Census Region and income level, MEPS survey data 2010-2017. Note: * P-value ≤ 0.05, ** p-value ≤ 0.01, and *** p-value ≤ 0.001.

Changes in reported insurance coverage associated with the ACA by income for non-elderly individuals with any of the MEPS chronic conditions, by region, are shown in Fig 1. As expected, given the focus of the ACA on low-income individuals, gains in insurance coverage for those in low- and very low-income levels were higher than gains in other income levels within all regions. Comparing regions, gains in insurance coverage for those in the very low-income bracket were higher in the Midwest, and West relative to in the South (11.89%, 14.52 % vs. 8.99%). Moreover, all regions exhibited increases in insurance coverage for those in the low-income bracket (Northeast 9.85%, Midwest 9.94%, West 16.76%, and South 8.54%) and middle-income bracket (Northeast 3.70%, Midwest 6.65%, West 9.18%, and South 4.48%). The changes for the high-income brackets were modest in all regions, though positive (Northeast 1.60%, Midwest 1.41%, West 3.01%, and South 1.82%).

## Discussion

After ACA implementation in January 2014, uninsurance rates among non-elderly adults declined sharply from 20.4% in 2013 to 12.8% by the end of 2015 nationwide, notably for lower-income populations [14,15]. Our findings demonstrated that non-elderly adults with one or more self-reported chronic condition—the segment of the population arguably most poised to benefit from gaining health insurance—reported substantial increases in insurance coverage. Nevertheless, there were sizeable differences in these coverage gains by region and income. The largest gains in insurance access were realized in the West region. The South region, which had a notably higher prevalence of chronic conditions pre-ACA, experienced the smallest increase in insurance coverage for those with chronic conditions relative to other regions. We found that access to health insurance coverage improved in all but the highest income bracket after the ACA.

Despite these substantial gains in coverage for clinically vulnerable Americans, lack of insurance coverage remains meaningful in all regions after the ACA—and most notable in the South. For example, an individual with diabetes living in the South was 1.99 times more like to be uninsured compared to a similar individual in the Northeast, the region with the highest access both before and after the ACA. For non-elderly adults with ≥ 2 chronic conditions, South region residents had 2.29 times the likelihood of being uninsured compared to Northeast region residents.

The increases in coverage after the ACA among adults with self-reported chronic conditions were likely driven by the combination of improved affordability of private coverage on the exchanges, Medicaid expansion, and the market protections such as guaranteed insurance access despite preexisting conditions. Notably, very low-income, low-income, and middle-income adults in the South still reported increased insurance coverage, despite little Medicaid expansion and health exchanges that were not well supported. Improved access for low-income Americans in states with Medicaid expansion has been associated with lower mortality [16] A similar benefit for low-income patients cared for at Federally Qualified Health Centers with hypertension has also been documented [17]. The increase in reported access to health insurance coverage that we report for those with chronic conditions would be expected to have similar health benefits.

## Limitations

This study has several limitations. First, we examined ACA-associated changes among patient cohorts with chronic conditions separately, and these cohorts may be overlapping. For example, the population defined as having “heart disease/stroke” are likely to have hypertension and/or diabetes. To address this limitation, we performed two separate sensitivity analyses within each self-reported chronic condition cohort (Appendix A and B): 1) for patients with only the specified chronic condition and 2) for patients with the specified chronic condition who have at least one other condition. These sensitivity analyses showed similar patterns of increases in insurance coverage for those with at least one other condition across all chronic condition cohorts. In the West, individuals the select chronic conditions had the highest increase in reported insurance coverage post-ACA (high blood pressure +7.69%, heart disease/stroke +8.69%, asthma +8.51%, and arthritis +8.75%). The South region showed a much lower reported increase in health insurance access post-ACA for individuals with diabetes, heart disease/stroke, asthma, and arthritis compared to Midwest and West (diabetes: +4.93% vs Midwest +8.6%, West +8.32%; heart disease/stroke: +5.28% vs Midwest +7.58% and West +8.69%; and asthma: +5.28% vs. Midwest +6.82% and West +8.51%) (Appendix A). The result of the sensitivity analysis for the mutually exclusive patient cohort (patients with the only specified condition) was consistent with our main results that in the West, respondents with only diabetes, hypertension, or asthma had improved access to insurance post-ACA (diabetes: +17.78%, high blood pressure: +9.26%, and asthma: +10.34%). However, the sensitivity analysis results were not statistically significant in the South region for diabetes, heart disease, and asthma (Appendix B).

Second, the MEPS chronic condition diagnostic information was self-reported by a household respondent and thus subject to recall and other biases. The MEPS captures individual-level experiences and perceptions regarding health and insurance access and thus offers a granular view of the respondent’s basis for health-related decision-making. Our data lacked additional respondent-level information at the zip-code, county, or state level that would allow more income and geographically precise covariates. Our findings are restricted to the regional level and are generalizable based on regional differences, in this care the much lower levels of Medicaid expansion in the South. Third, our results may not extend to populations not represented in the MEPS such as undocumented immigrants. Fourth, our analysis was further limited to the self-reported chronic conditions represented in the MEPS, which is a subset of the conditions identified by the U.S Department of Health and Human Services for chronic disease research [18]. Some chronic conditions may not have been reported. Fifth, there could be differences in within-region factors that explain why one region would see greater increases in insurance coverage than other regions as a result of the ACA. These factors may relate to resources in state Medicaid agencies devoted to outreach to the uninsured, availability of people who help the uninsured find and select coverage, and other social or cultural differences in addition to the difference in the political affiliation of a state’s governmental leadership. Finally, our statistical analyses produced associations that do not imply causality, as there may have been contemporaneous changes in sampling, respondent behavior, or social or environmental circumstances that were associated with both the post-ACA period and with insurance coverage.

## Conclusions

Health insurance coverage among non-elderly adults with self-reported chronic conditions increased after the ACA across all US regions and all income levels. The increase was largest in the West and smaller in the Northeast and South regions, the lower change in the Northeast being attributable to the higher pre-ACA levels of health insurance access.

Our data suggest an important “ripple effect” of the ACA package of reforms across all levels of income as demonstrated by the increase in insurance access reported by non-elderly adults with multiple self-reported chronic conditions nationwide. Most importantly, the increase in insurance access for those with self-reported chronic conditions was highest for lower-income non-elderly adults. This was apparent even in the South, albeit to a lesser degree, where the adoption of ACA Medicaid expansion has been relatively limited.

The considerably lower access to health insurance access reported by non-Medicare individuals who have any chronic condition in the US South region is a hardship that transcends racial, ethnic, income, social, and sexual orientation categories. Unfortunately, state-by-state decisions to capitalize on federal incentives to expand Medicaid have been constrained by many forces, some related to political opposition to federal policy making and some related to other factors such as competing budgetary demands. Greater political consensus or increased political will among policymakers in the South US census region are likely needed to move toward national parity of access to health insurance coverage for those with chronic illnesses. Ultimately, more will need to be known about all the factors that contribute to a state’s reluctance to capitalize on the ACA opportunities. Given the intrinsic health needs of the population with chronic diseases and the high prevalence, especially in the US South, regional differences we show among individuals who are otherwise very similar should serve to stimulate further exploration of barriers to expansion and potential solutions.

Access to health insurance coverage remains an unfulfilled national need. There have been substantial gains in reported access across all US regions, but there is more work ahead, even in those states that have fully embraced the ACA. Current proposals to provide additional federal incentives for states to expand Medicaid offer an important opportunity to extend the benefits of insurance coverage in states that have not yet expanded Medicaid [19]. Our data offers a stark comparison between the South and the rest of the US. For the sake of the many who live with one or more chronic condition, efforts to expand access demand citizen and governmental action.

## Supporting information

S1 File (DOCX)
