# Supplementary material for: Changes in coverage among non-elderly adults with chronic diseases following Affordable Care Act implementation
Source: PLoS One. 2022 Nov 30;17(11):e0278414. doi: 10.1371/journal.pone.0278414 (PMC9710786; doi:10.1371/journal.pone.0278414)
Supplement: S1 File — (DOCX) [file pone.0278414.s001.docx]

**Supporting material: Appendix A.**

Sensitivity Analysis of the Impact of ACA on Insurance Status of Individuals **with each Specified Chronic Condition and at least One Other Condition** Ages 18-64 by US Census Region,

MEPS Survey Data 2010-2017

| **Share of the Population with Insurance Coverage** | | | | | | | |  |  | |
| --- | --- | --- | --- | --- | --- | --- | --- | --- | --- | --- |
|  | Unadjusted | | |  | Adjusted Change | | | | | |
|  | Pre-ACA | Post-ACA | Percentage Point Change | Percentage Point Change | |  | p-value | | | |
| **Diabetes** |  |  |  |  | |  |  | | | |
| Northeast | 90.67 | 92.64 | +1.96 | +1.35 | |  | 0.363 | | | |
| Midwest | 83.55 | 92.41 | +8.86 | +8.76 | |  | <0.001 | | | |
| South | 78.23 | 84.13 | +5.91 | +4.93 | |  | <0.001 | | | |
| West | 79.74 | 89.02 | +9.28 | +8.32 | |  | <0.001 | | | |
| **High blood pressure** | | | | | | | | | |  |
| Northeast | 89.71 | 93.33 | +3.63 | +3.79 | |  | <0.001 | | | |
| Midwest | 84.62 | 91.79 | +7.16 | +6.88 | |  | <0.001 | | | |
| South | 77.19 | 83.42 | +6.23 | +6.23 | |  | <0.001 | | | |
| West | 81.83 | 90.23 | +8.40 | +7.69 | |  | <0.001 | | | |
| **Heart disease/ stroke** | | | | | | | | | |  |
| Northeast | 90.68 | 94.12 | +3.44 | +3.38 | |  | 0.003 | | | |
| Midwest | 85.13 | 92.78 | +7.65 | +7.58 | |  | <0.001 | | | |
| South | 79.62 | 85.30 | +5.68 | +5.28 | |  | <0.001 | | | |
| West | 83.72 | 92.79 | +9.06 | +8.69 | |  | <0.001 | | | |
| **Asthma** |  |  |  |  | |  |  | | | |
| Northeast | 91.01 | 94.81 | +3.80 | +4.00 | |  | 0.001 | | | |
| Midwest | 86.01 | 92.87 | +6.86 | +6.82 | |  | <0.001 | | | |
| South | 79.70 | 85.68 | +5.98 | +5.28 | |  | <0.001 | | | |
| West | 84.34 | 93.86 | +9.52 | +8.51 | |  | <0.001 | | | |
| **Arthritis** |  |  |  |  | |  |  | | | |
| Northeast | 91.84 | 94.93 | +3.09 | +2.95 | |  | <0.001 | | | |
| Midwest | 86.42 | 93.59 | +7.17 | +7.14 | |  | <0.001 | | | |
| South | 80.35 | 86.66 | +6.31 | +6.38 | |  | <0.001 | | | |
| West | 84.58 | 93.75 | +9.17 | +8.75 | |  | <0.001 | | | |

Note: We used separate logistic regression models for each chronic condition and

comorbidity adjusted for sex, age, race, family income, education, smoking status,

employment status, perceived physical and mental health status, and having

a usual source of care and present the percentage-point change in insurance coverage.

**Supporting material: Appendix B.**

Sensitivity Analysis of the Impact of ACA on Insurance Status of Individuals **with only Specified Chronic Condition** Ages 18-64 by US Census Region, MEPS Survey Data 2010-2017

| **Share of the Population with Insurance Coverage** | | | | | | | |  |  | |
| --- | --- | --- | --- | --- | --- | --- | --- | --- | --- | --- |
|  | Unadjusted | | |  | Adjusted Change | | | | | |
|  | Pre-ACA | Post-ACA | Percentage Point Change | Percentage Point Change | |  | p-value | | | |
| **Diabetes** |  |  |  |  | |  |  | | | |
| Northeast | 82.00 | 90.54 | +8.54 | +5.93 | |  | 0.387 | | | |
| Midwest | 81.18 | 87.23 | +6.06 | +9.88 | |  | 0.129 | | | |
| South | 66.02 | 70.15 | +4.13 | +3.24 | |  | 0.460 | | | |
| West | 62.58 | 83.95 | +21.37 | +17.78 | |  | <0.001 | | | |
| **High blood pressure** | | | | | | | | | |  |
| Northeast | 83.77 | 86.71 | +2.94 | +2.74 | |  | 0.209 | | | |
| Midwest | 80.11 | 86.85 | +6.73 | +0.94 | |  | 0.638 | | | |
| South | 72.49 | 77.59 | +5.10 | +4.79 | |  | 0.001 | | | |
| West | 73.79 | 82.21 | +8.42 | +9.26 | |  | <0.001 | | | |
| **Heart disease/ stroke** | | | | | | | | | |  |
| Northeast | 85.86 | 92.39 | +6.52 | +8.30 | |  | 0.011 | | | |
| Midwest | 73.54 | 82.19 | +8.64 | +12.89 | |  | <0.001 | | | |
| South | 70.88 | 77.57 | +6.69 | +4.68 | |  | 0.156 | | | |
| West | 78.55 | 89.74 | +11.19 | +10.46 | |  | 0.003 | | | |
| **Asthma** |  |  |  |  | |  |  | | | |
| Northeast | 85.88 | 90.41 | +4.53 | +5.39 | |  | 0.023 | | | |
| Midwest | 81.00 | 89.88 | +8.87 | +5.36 | |  | 0.014 | | | |
| South | 71.25 | 83.09 | +11.84 | +4.17 | |  | 0.072 | | | |
| West | 79.86 | 90.76 | +10.90 | +10.34 | |  | <0.001 | | | |
| **Arthritis** |  |  |  |  | |  |  | | | |
| Northeast | 80.93 | 91.03 | +10.10 | +6.88 | |  | 0.071 | | | |
| Midwest | 80.46 | 88.75 | +8.29 | +11.94 | |  | <0.001 | | | |
| South | 68.26 | 80.16 | +11.90 | +7.82 | |  | 0.012 | | | |
| West | 82.72 | 90.11 | +7.39 | +3.09 | |  | 0.344 | | | |

Note: We used separate logistic regression models for each chronic condition and

comorbidity adjusted for sex, age, race, family income, education, smoking status,

employment status, perceived physical and mental health status, and having

a usual source of care and present the percentage-point change in insurance coverage.
